# Single catheter primary percutaneous coronary intervention method in patients with ST-elevation myocardial infarction: the SPEEDY-PCI study

**DOI:** 10.1007/s12928-025-01162-1

**Published:** 2025-07-07

**Authors:** Sho Torii, Akihiko Takahashi, Yujiro Ono, Masanori Taniwaki, Mitsutoshi Oguri, Masanori Teramura, Ryuichi Kato, Shuji Otsuki, Hiroshi Suzuki, Fuminobu Yoshimachi, Hironori Ueda, Keisuke Shioji, Gaku Nakazawa, Kaoru Sakurai, Mitsuru Tsujimoto, Motosu Ando, Toshiyuki Kozai, Rie Aoyama, Yuji Ikari

**Affiliations:** 1 Department of Cardiology, Tokai University School of Medicine, 143 Shimokasuya, Isehara, Kanagawa 259-1193 Japan; 2 Department of Cardiology, Sakurakai Takahashi Hospital, Kobe, Japan; 3 Department of Cardiology, HigashiHiroshima Medical Center, Hiroshima, Japan; 4 Department of Cardiology, Tokorozawa Heart Center, Saitama, Japan; 5 Department of Cardiology, Kasugai Municipal Hospital, Kasugai, Aichi Japan; 6 Department of Cardiology, Ichinomiya Nishi Hospital, Ichinomiya, Aichi Japan; 7 Department of Cardiology, Social Medical Corporation Yamatokai Foundation Higashiyamato Hospital, Tokyo, Japan; 8 Department of Cardiology, Sonoda Daiichi Hospital, Tokyo, Japan; 9 Department of Cardiology, Showa Medical University Fujigaoka Hospital, Yokohama, Kanagawa Japan; 10 Department of Cardiology, Tokai University Hachioji Hospital, Tokyo, Japan; 11 Department of Cardiology, Hiroshima Prefectural Hospital, Hiroshima, Japan; 12 Department of Cardiology, Kishiwada City Hospital, Osaka, Japan; 13 Department of Cardiology, Kindai University Hospital, Osaka, Japan; 14 Department of Cardiology, Shinyurigaoka General Hospital, Kanagawa, Japan; 15 Department of Cardiology, The Veritas Hospital, Kawanishi, Hyogo Japan; 16 Department of Cardiology, Okamura Memorial Hospital, Shizuoka, Japan; 17 Department of Cardiology, Munakata Suikokai General Hospital, Fukuoka, Japan; 18 Department of Cardiology, Funabashi Municipal Medical Center, Chiba, Japan

**Keywords:** Primary percutaneous coronary intervention, Ikari curve, ST-elevation myocardial infarction, Single-catheter PCI

## Abstract

Reducing total ischemic time in ST-elevation myocardial infarction (STEMI) is crucial for improving outcomes. While procedural time during primary percutaneous coronary intervention (PCI) is critical, strategies to shorten it need to be explored. To examine whether the single-catheter PCI (SC-PCI) method using a universal guiding catheter, Ikari-Left curve, without catheter exchange reduces PCI time compared to conventional PCI (C-PCI). The Speedy PCI study is a prospective, multicenter, randomized trial comparing SC-PCI versus C-PCI. The primary endpoint was the time from sheath insertion to first device activation (S2B) time. Secondary endpoints included door-to-balloon time, total ischemia time, fluoroscopy time, contrast volume, hospital expenses, and 30-day mortality. A total of 380 patients (SC-PCI: 194; C-PCI: 186) were analyzed. Both groups had high primary PCI success rates (92.3 vs. 91.9%, *p* = 0.74) and similar radial access usage (higher than 97%). SC-PCI method showed a significantly shorter S2B time (15.8 ± 10.9 min) compared to the C-PCI method (18.7 ± 10.6 min, *p* = 0.007) with reduced number of total catheters used (1.2 ± 0.6 vs. 2.7 ± 0.7, *p* < 0.0001, respectively). No cases of coronary dissection caused by the guiding catheter were observed in the SC-PCI method. Clinical outcomes at 30 days and 1-year follow-up, including all-cause mortality and stroke rates, were similar between the groups. The SC-PCI method using the Ikari curve demonstrated a significant reduction in PCI procedure time while maintaining safety and primary PCI success in primary PCI for STEMI.

**Graphical abstract:**

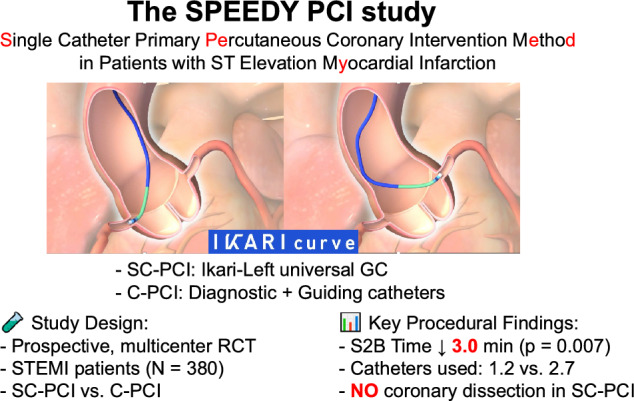

**Supplementary Information:**

The online version contains supplementary material available at 10.1007/s12928-025-01162-1.

## Introduction

Reducing total ischemic time with a primary percutaneous coronary intervention (PCI) is a gold standard for improving mortality in patients with ST-elevation myocardial infarction (STEMI) [1, 2]. Several studies have been conducted to shorten the total ischemic time by minimizing the duration from the onset of STEMI to arrival at the catheterization lab. Strategies include raising public awareness about STEMI symptoms and the importance of early response, utilizing electrocardiogram transmission systems from ambulance services to the hospitals [3–8], improving in-hospital announcements, and enhancing professional team training [9–11]. However, reducing sheath-to-balloon (S2B) time, defined as the time between sheath insertion and first device activation, has not been well studied. S2B time reflects pure procedural efficiency within the catheterization laboratory, unaffected by external factors, such as ambulance logistics or emergency room protocols. By targeting S2B time as the primary endpoint, we aimed to isolate the procedural impact of the SC-PCI method on STEMI care.

The universal guiding catheter, including the Ikari-Left (IL) catheter, is designed to engage both the right and left coronary arteries with a single guiding catheter. The safety and feasibility of the IL guiding catheter have been demonstrated in an observational study involving 621 consecutive cases [12]. We recently performed retrospective observational studies to assess the feasibility and safety of the single universal guiding catheter PCI (SC-PCI) method, which employs a universal guiding catheter from the beginning of angiography [13, 14]. The results demonstrated that the SC-PCI method reduced both door-to-balloon (D2B) time and S2B time by allowing operators to perform both angiography and primary PCI for the right and left coronary arteries using a single universal guiding catheter. However, the efficacy of the SC-PCI method was not well evaluated due to the retrospective nature of the analysis and the limited number of patients. Therefore, the single catheter primary percutaneous coronary intervention method in patients with ST-elevation myocardial infarction (SPEEDY-PCI) study was conducted as a multicenter, prospective, randomized controlled.

## Methods

### Study design and population

The SPEEDY-PCI study was a physician-initiated, prospective, multicenter, open-label, randomized clinical trial conducted in Japan from November 2022 to October 2023. It aimed to evaluate the efficacy of the SC-PCI method in primary PCI compared to the conventional (C-PCI) method. The study protocol specified the use of biodegradable polymer-coated everolimus-eluting stents (Synergy, Boston Scientific, MA, USA) in all cases. Informed consent was obtained from patients prior to coronary angiography. In cases where written informed consent could not be obtained due to severe clinical conditions, verbal consent was obtained from the patients or their legal representatives, followed by written consent after patient recovery.

Patients with STEMI who were eligible for primary PCI within 12 h of symptom onset were randomly assigned in a 1:1 ratio to either the SC-PCI or C-PCI group. Randomization was conducted using a secure, web-based randomization system managed independently by the data center. Exclusion criteria included patients under 21 years old, those in cardiogenic shock requiring mechanical support (such as Impella, extracorporeal membrane oxygenation, and percutaneous cardiopulmonary support), patients with bradycardia requiring a temporary pacemaker, hemodialysis, and other conditions deemed ineligible for the study. Patients who did not require mechanical circulatory support or temporary pacemaker placement at the time of randomization but subsequently required such interventions during the primary PCI procedure, were included in the study population. The sponsor was not involved in any part of the study's conduct. This study adhered to the Declaration of Helsinki principles for human research, and the Tokai University certified review board approved the study protocol. This trial was registered under ClinicalTrials.gov number: NCT05604976.

### The SC-PCI method and the C-PCI method

The operators in the study were required to have experience in treating at least five cases of both right coronary artery (RCA) and left coronary artery (LCA) using Ikari-Left (IL) guiding catheters from radial artery access.

In the SC-PCI method, primary PCI begins with a universal guiding catheter (IL) used for diagnostic coronary angiography to identify the culprit vessel causing STEMI. The procedure starts with the angiography of the non-culprit coronary artery, as predicted by electrocardiogram and/or echocardiogram, followed by angiography of the culprit coronary artery. If the diagnosis of the culprit vessel is confirmed, primary PCI is performed directly without changing catheters. This method allows operators to skip steps, such as engaging diagnostic catheters and the insertion and removal of diagnostic catheters.

In the C-PCI method, initial diagnostic angiography is performed using diagnostic catheters. Following this, primary PCI was performed by replacing the diagnostic catheter with a guiding catheter. Various approaches can be used for diagnostic angiography, including separate catheters for the right and left coronary arteries, dual-purpose contrast catheters, or a combination of one contrast catheter and a guiding catheter. Any of these methods is acceptable as long as the procedure begins with a diagnostic catheter. After diagnostic catheter use, the choice of guiding catheter is at the operator’s discretion, meaning that the IL guiding catheter was also available for primary PCI within the C-PCI method.

### PCI procedure and medications

As the study was conducted during the COVID-19 era, decisions regarding primary PCI for STEMI patients, such as whether to proceed with the procedure after confirming antigen test results, were left to the discretion of each participating facility's policy. In addition, the choice of antiplatelet and anticoagulant therapy before enrollment, as well as the dosage of intravenous heparin used before and after PCI, was left to the discretion of the investigators. Success of primary PCI was defined as achieving a thrombolysis in myocardial infarction (TIMI) 3 blood flow in the culprit coronary artery at the end of the procedure.

All STEMI patients received dual antiplatelet therapy (DAPT). If the patients had not taken any antiplatelet drugs, they were administered a loading dose of 200 mg to 300 mg aspirin and a P2Y12 inhibitor. Procedural anticoagulation was achieved with an initial bolus of unfractionated heparin. A supplemental bolus was administered during the procedure to maintain an activated clotting time of over 250 s. The approach site, sheath size, type of diagnostic, and guiding catheters used in the C-PCI method, the use of intracoronary imaging devices, and the duration of DAPT after primary PCI were all determined at the operators'discretion.

### Endpoints

The primary endpoint was S2B time, defined as the time between sheath insertion and first therapeutic device activation such as aspiration catheter, balloon, and excimer laser catheter.

The secondary endpoints at admission comprised D2B time, defined as the interval from the patient's arrival at the emergency department to the first device activation, total ischemic time, contrast dye volume, radiation exposure dose, fluoroscopic time, the number of diagnostic and guiding catheters used, procedural success of the SC-PCI method, admission fees, predictors of faster S2B time, and S2B time by institute. Total ischemic time was defined as the time from symptom onset to first device activation.

The secondary endpoints at 30-day follow-up included all-cause death, cardiac death, and hemorrhage. The secondary endpoints at 1-year follow-up included all-cause death and cardiac death. Hemorrhage was defined as bleeding academic research consortium (BARC) type 3 or 5 bleeding events [15].

The independent clinical event committee adjudicated all the clinical events comprising the coprimary end points based on the source documents in a blinded fashion to the assigned treatment groups.

### Definitions

Primary PCI success was defined as achieving final TIMI 3 flow in the culprit artery after PCI. The SC-PCI method success was defined as completing the procedure with a single Ikari-Left catheter High bleeding risk was classified according to ARC-HBR criteria [16].

### Statistical analysis

Sample size calculation was performed on the basis of the previous multicenter retrospective study [13]. The study demonstrated that S2B of the SC-PCI method vs. the C-PCI method was 19 ± 14 min vs. 23 ± 14 min (*p* < 0.0001). Therefore, when testing the level of significance of 0.05 and the power of 0.8 based on the value, the number of patients calculated was 388 cases. Considering 3% dropout cases, a total of 400 patients were initially planned. However, in the blinded monitoring of the 300 patients initially enrolled, the number of patients with MI with non-obstructive coronary arteries (MINOCA) was higher than expected. The clinical study managers and statistician revised the sample size calculation, resulting additional enrollment of 30 patients in the study.

Continuous normally distributed variables were expressed as mean ± standard deviation and analyzed using Student’s *t* tests. Categorical variables were expressed as numbers and percentages, analyzed using the Chi-square test. The analysis for predictors of D2B time and sheath-to-balloon time in each institute used the general linear model.

Linear regression analysis was performed to examine factors associated with S2B time. Note that the SC-PCI method, the primary endpoint of this study, was excluded from both the univariate and multivariate analyses to avoid confounding effects. Multivariate analysis was performed using factors that were found to be significantly different in univariate analysis. Finally, a *p* value of < 0.05 was considered statistically significant. All statistical analyses were performed using SAS version 9.4 (SAS Institute, Cary, North Carolina).

## Results

### Patients’ recruitment and assignment

**Fig. 1:**
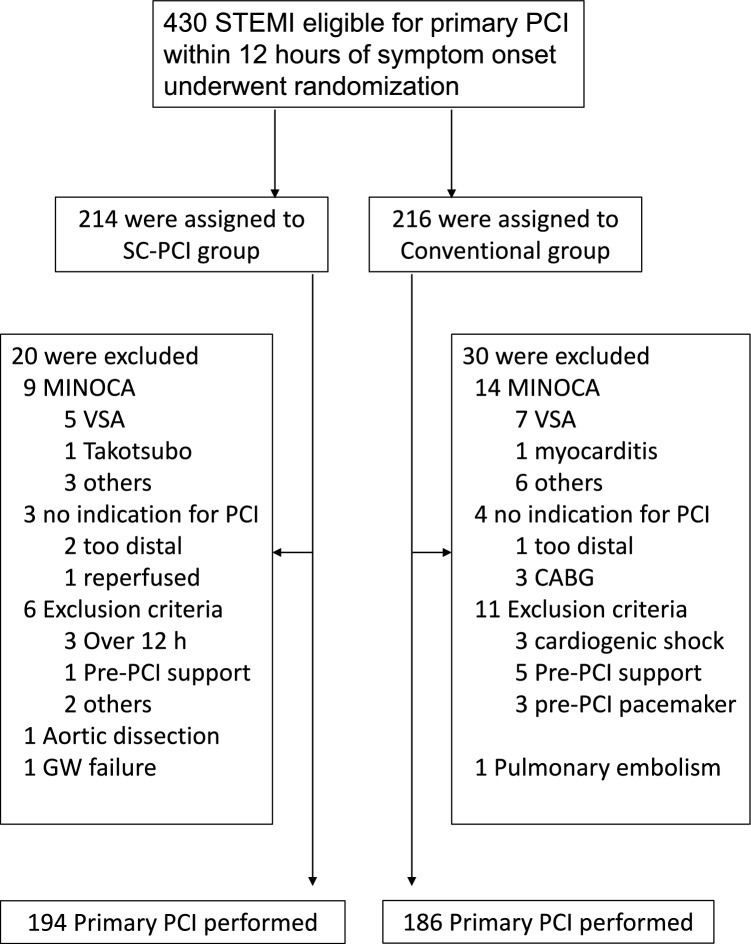
Randomization of patients *STEMI* ST-elevation myocardial infarction, *SC-PCI* single universal catheter percutaneous coronary intervention, *MINOCA* myocardial infarction with non-obstructive coronary artery, *VSA* vaso-spastic angina, *PCI* percutaneous coronary intervention, *CABG* coronary artery bypass graft, *GW* guide wire

In total, 430 patients were enrolled and randomly assigned from 47 centers in Japan (Supplemental Table). The full analysis included 194 patients in the SC-PCI method and 186 patients in the C-PCI method, after excluding 20 patients from the SC-PCI method and 30 from the C-PCI method. The reasons for exclusion were patients with MINOCA, aortic dissection, pulmonary embolism, and those without an indication for primary PCI. Additionally, 16 patients (6 from the SC-PCI method and 11 from the C-PCI method) met exclusion criteria such as the need for a temporary pacemaker before PCI (Fig. 1).

### Baseline characteristics

The baseline characteristics were well balanced between the 2 groups, except for higher heart rate at admission in the SC-PCI method compared with the C-PCI method. The mean age was 69 ± 13 years, and 81.6% of patients were men (Table 1).

**Table 1:** Patient characteristics

	SC-PCI group *N* = 194	Conventional group *N* = 186	Total *N* = 380	*p* value
Age—years	69 ± 12	69 ± 13	69 ± 13	0.91
Male sex—no.(%)	159 (82.0)	151 (81.2)	310 (81.6)	0.85
Height—cm	164 ± 8	164 ± 9	164 ± 9	0.94
Body weight—kg	65 ± 13	66 ± 13	65 ± 13	0.89
BMI—kg/m^2^	24 ± 4	24 ± 4	24 ± 4	0.81
Smoking current or past—%	130 (67)	115 (62)	245 (64)	0.36
Diabetic mellitus—no.(%)	66 (34)	46 (25)	112 (29)	0.14
Dyslipidemia—no.(%)	125 (64)	126 (68)	251 (66)	0.79
Hypertension—no.(%)	130 (67)	133 (72)	263 (69)	0.63
Prior PCI—no.(%)	20 (10)	23 (12)	43 (11)	0.53
Prior CABG—no.(%)	0	0	0	
Old myocardial infarction—no.(%)	12 (6)	17 (9)	29 (8)	0.28
Prior ischemic stroke, %	8 (4.1)	6 (3.2)	14 (3.7)	0.65
Prior hemorrhagic stroke—no.(%)	1 (0.5)	3 (1.6)	4 (1.1)	0.29
Prior heart failure—no.(%)	0	2 (1.1)	2 (0.5)	0.15
Lower extremity artery disease—no.(%)	6 (3.1)	3 (1.6)	9 (2.4)	0.40
History of cancer—no.(%)	4 (2.1)	6 (3.2)	10 (2.6)	0.47
Oral anticoagulant—no.(%)	7 (3.6)	9 (4.8)	16 (4.2)	0.55
High bleeding risk—no. (%)	59 (30.4)	64(34.4)	123 (32.4)	0.47
Killip classification I/II/III/IV, %	78.4/17.0/2.6/2.1	81.2/14.0/3.8/1.1	79.8/15.5/3.2/1.6	
Systolic blood pressure, mmHg	144 ± 28	141 ± 28	142 ± 28	0.09
Diastolic blood pressure, mmHg	89 ± 20	86 ± 19	87 ± 19	0.33
Heart rate,/min	81 ± 17	76 ± 16	79 ± 17	0.54
Creatinine, mg/dL	0.9 ± 0.3	0.9 ± 0.4	0.9 ± 0.4	0.27
eGFR, ml/min/1.73 m^2^	68 ± 22	65 ± 19	66 ± 21	0.31
Hemoglobin, g/dL	14.2 ± 2.1	14.1 ± 1.9	14.2 ± 2.0	
Platelet count, × 10^4^/mL^3^	22.4 ± 6.3	23.1 ± 6.5	22.8 ± 6.4	0.67
Grace risk score	156 ± 32	155 ± 31	155 ± 31	0.65

*BMI* body mass index, *eGFR* estimated glomerular filtration rate, *GRACE* global registry of acute coronary events, *PCI* percutaneous coronary intervention, *CABG* coronary artery bypass graft, *SC-PCI* single-catheter percutaneous coronary intervention

As the study was conducted during the COVID-19 era (November 2022 to October 2023), there was variability between facilities in handling patients upon arrival at the hospital. Most facilities began primary PCI after submitting an antigen test and implementing infection control measures, without waiting for the antigen test results. However, some facilities performed chest CT scans to determine if patients had COVID-19 pneumonia, while others waited for the COVID-19 antigen test results before proceeding to the cath lab.

### PCI procedures

Radial or distal-radial access was used in 97.4% of cases, with 322 cases (87%) performed via right radial access and 48 cases (13%) via left radial access, with similar prevalence between the 2 groups. The SC-PCI method demonstrated an 88.1% success rate, enabling both diagnostic angiography and PCI with a single Ikari-Left guiding catheter, eliminating the need for catheter exchange. The number of diagnostic catheter (0.9 ± 0.4 vs. 1.7 ± 0.6, respectively, *p* < 0.0001) was significantly less in the SC-PCI method compared with the C-PCI method. On the other hand, the number of guiding catheter was similar between the 2 groups (1.1 ± 0.3 vs. 1.1 ± 0.4, respectively, *p* = 0.86). Type of guiding catheter was different with higher prevalence of Ikari catheter usage (94.9 vs. 40.9%, respectively) (Table 2, Supplemental Table 2). Coronary dissection caused by guiding catheters was 1 case from the C-PCI method and none in the SC-PCI method (Table 3).

**Table 2:** Procedural characteristics

	SC-PCI group *N* = 194	Conventional group *N* = 186	Total *N* = 380	*p* value
Access site				
Radial/distal radial—no. (%)	189 (97.4)	181 (97.3)	370 (97.4)	0.61
Radial—no.(%)	161 (83.0)	146 (78.5)	307 (80.8)	
Distal radial—no.(%)	28 (14.4)	35 (18.8)	63 (16.6)	
Femoral—no.(%)	4 (2.1)	3 (1.6)	7 (1.8)	
Other—no.(%)	1 (0.5)	2 (1.1)	3 (0.8)	
Access change -no.(%)	8 (4.1)	6 (3.2)	14 (3.7)	0.64
Culprit lesion				
RCA—no.(%)	63 (32.5)	69 (37.1)	133 (35.0)	0.18
LAD—no.(%)	118 (60.8)	100 (53.8)	217 (57.1)	
LCX—no.(%)	11 (5.7)	17 (9.1)	28 (7.4)	
LM—no.(%)	2 (1.0)	0	2 (0.5)	
No. of diseased vessel				
1—no.(%)	129 (66.5)	118 (63.4)	247 (65.0)	0.63
2—no.(%)	45 (23.2)	43 (23.1)	88 (23.2)	
3—no.(%)	20 (10.3)	25 (13.4)	45 (11.8)	
Non-protected left main disease	7 (3.6)	3 (1.6)	10 (2.6)	0.22
No. of angiographic catheter	0.9 ± 0.4	1.7 ± 0.6	0.9 ± 0.9	< 0.0001
No. of guiding catheter	1.1 ± 0.3	1.1 ± 0.4	1.1 ± 0.3	0.86
Total no. of catheter used	1.2 ± 0.6	2.7 ± 0.7	2.0 ± 1.0	< 0.0001
Guiding catheter shape				< 0.0001
Ikari—no.(%)	184 (94.9)	76 (40.9)	261 (68.7)	
Judkins—no.(%)	6 (3.1)	44 (23.7)	49 (12.9)	
Amplatz—no.(%)	1 (0.5)	10 (5.4)	11 (2.9)	
VODA/EBU/XB—no.(%)	2 (1.0)	47 (25.2)	49 (12.9)	
Others—no.(%)	1 (0.5)	9 (4.8)	10 (2.6)	
Sheath size				0.48
Sheathless (6F)—no.(%)	18 (9.3)	24 (12.9)	42 (11.0)	
5F—no.(%)	3 (1.6)	1 (0.5)	4 (1.1)	
6F—no.(%)	160 (82.5)	146 (78.5)	306 (80.5)	
7F—no.(%)	13 (6.7)	15 (8.1)	28 (7.4)	
Guiding catheter size				
5F—no.(%)	2 (1.0)	1 (0.5)	3 (0.8)	0.32
6F—no.(%)	184 (94.9)	171 (91.9)	355 (93.4)	
7F—no.(%)	8 (4.1)	14 (7.5)	22 (5.8)	
Post-PCI pacemaker—no.(%)	3 (1.6)	4 (2.2)	7 (1.8)	0.66
Post-PCI IABP—no.(%)	10 (5.2)	8 (4.3)	18 (4.7)	0.70
Post-PCI ECMO—no.(%)	2 (1.0)	2 (1.1)	4 (1.1)	0.96
Post-PCI Impella—no.(%)	1 (0.5)	0	1 (0.3)	0.33

High bleeding risk was defined according to the academic research consortium for high bleeding risk (ARC-HBR) criteria

*LAD* left anterior descending artery, *LCX* left circumflex artery, *RCA* right coronary artery, *LM* left main, *IABP* intra-aortic balloon pump, *ECMO* extracorporeal membrane oxygenation, *F* french size (catheter size), *PCI* percutaneous coronary intervention

**Table 3:** PCI results

	SC-PCI group *N* = 194	Conventional group *N* = 186	Total *N* = 380	*p* value
Primary PCI success—no.(%)	179 (92.3)	171 (91.9)	350 (92.1)	0.90
SC-PCI method success—no.(%)	171 (88.1)	–	–	
Sheath-to-balloon time, min	15.8 ± 10.9	18.7 ± 10.6	17.2 ± 10.9	0.007
Door to sheath time, min	49.4 ± 36.2	44.1 ± 21.1	46.8 ± 29.8	0.08
Door-to-balloon time, min	65.2 ± 38.2	62.8 ± 23.4	64.0 ± 31.8	0.47
Onset-to-door time, min	171.6 ± 152.1	179.4 ± 150.8	175.4 ± 151.3	0.61
Onset-to-balloon time, min	236.8 ± 157.4	242.2 ± 154.0	239.5 ± 155.5	0.73
Total ischemic time, min	246.8 ± 163.7	245.2 ± 154.7	246.0 ± 159.1	0.92
Fluoroscopic time, min	22.0 ± 15.2	24.8 ± 18.2	23.4 ± 16.8	0.11
Radiation dose, mGy	1107 ± 811	1196 ± 952	1150 ± 883	0.33
Contrast dye volume, mL	132 ± 53	131 ± 52	132 ± 52	0.75
ICU stay, days	3.3 ± 3.5	3.1 ± 2.7	3.2 ± 3.1	0.58
Hospital stay, days	11.8 ± 8.6	12.5 ± 7.5	12.1 ± 8.1	0.39
Cost, million Yen	1.86 ± 1.23	1.78 ± 0.72	1.82 ± 1.0	0.44
Peak CK, IU/L	2623 ± 2278	2745 ± 2476	2682 ± 2451	0.63
Coronary dissection caused by the guiding catheter	0/194 (0)	1/186 (0.5)	1/380 (0.3)	0.3
Ikari curve—no./total no.(%)	0/185 (0)	0/76 (0)	0/261 (0)	0.31
Others—no./total no.(%)	0/9 (0)	1/110 (0.9)	1/119 (0.8)	

*PCI* percutaneous coronary intervention, *TIMI* thrombolysis in myocardial infarction, *S2B* sheath-to-balloon, *D2B* door-to-balloon, *CK* creatine kinase, *ICU* intensive care unit, *IL* Ikari Left (guiding catheter)

**Fig. 2:**
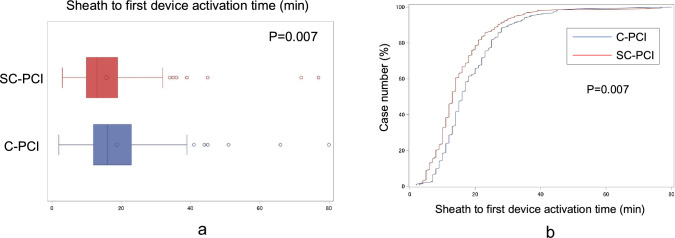
Sheath-to-balloon time in the SC-PCI method and the C-PCI method **A** Sheath-to-balloon time was significantly shorter in the SC-PCI method compared to the C-PCI method. **B** Distribution of S2B time in a cumulative frequency diagram of the SC-PCI and C-PCI groups *SC-PCI* single universal catheter percutaneous coronary intervention, *C-PCI* conventional percutaneous coronary intervention

S2B time, which is the primary endpoint of the study, was significantly shorter in the SC-PCI method compared to the C-PCI method with 3-min reduction of S2B time (15.8 ± 10.9 min vs. 18.7 ± 10.6 min, respectively, *p* = 0.007) (Fig. 2A,B, Table 3). On the other hand, the other secondary endpoints, including D2B time, onset to door time, and total ischemic time, were similar between the 2 groups. Fluoroscopic time and dose, as well as volume of contrast was also similar between the 2 groups (Table 3). Success rate of the SC-PCI method was 88.1% defined as completing the whole primary PCI procedure using only IL guiding catheter(s), 88.1% defined as completing the whole primary PCI procedure using an only single IL guiding catheter, and 85.1% defined as completing the whole primary PCI procedure using an only single IL guiding catheter with final TIMI 3 flow.

### Predictors for faster S2B time

Right radial access demonstrated a trend toward faster S2B time compared with left radial access (16.5 ± 9.5 min vs. 20.2 ± 14.9 min, respectively, p = 0.1); however, the difference was not statistically significant.

Univariate linear regression analysis showed that the following factors were significantly associated with S2B; SC-PCI method, Ikari curve, total catheter numbers, access change, hospital, radial access, and primary PCI success (Table 4). In the multivariate linear regression analysis using these factors, the model selected the following factors, SC-PCI method, total catheter numbers, hospital, and access change.

**Table 4:** Regression analysis for S2B time

	Univariate analysis					Multivariate analysis				
	F	B	Standard error	t	P	F	B	Standard error	t	P
SC-PCI method	7.29	2.98	1.105	2.70	0.007	6.54	−5.61	2.19	−2.56	0.011
Total catheter numbers	18.37	−65.88	9.823	−6.71	< 0.0001	9.17	−45.75	9.92	−4.61	< 0.0001
Hospital	2.93	−11.25	8.464	−2.09	< 0.0001	2.77	−4.31	7.56	7.03	< 0.0001
Access change	78.57	23.88	2.694	8.86	< 0.0001	32.01	−15.6	2.76	−5.66	< 0.0001
Radial/distal radial access	5.20	7.89	3.461	2.28	0.023					
Ikari curve	13.17	−4.28	1.180	−3.63	0.0003					
Primary PCI success	9.67	−12.74	4.096	−3.11	0.002					

*SC-PCI* single-catheter percutaneous coronary intervention, *C-PCI* conventional percutaneous coronary intervention, *S2B* Sheath-to-balloon, *PCI* Percutaneous coronary intervention, *B* Coefficient, *F* F-statistic

**Fig. 3:**
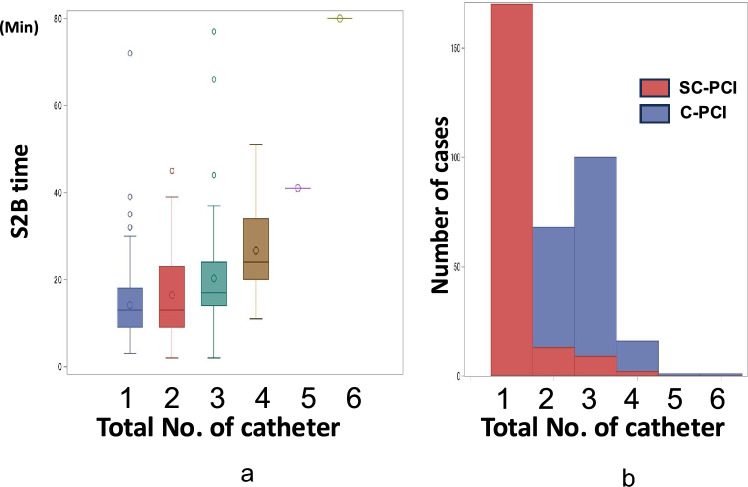
Relationship between total number of catheters and S2B time (**A**) and differences in SC-PCI and C-PCI groups (**B**). **A** S2B time increased as the total number of catheters increased, highlighting the importance of minimizing catheter usage in primary PCI. **B** In the SC-PCI group, the use of a single catheter was more prominent, whereas the use of three catheters was more common in the C-PCI group

**Fig. 4:**
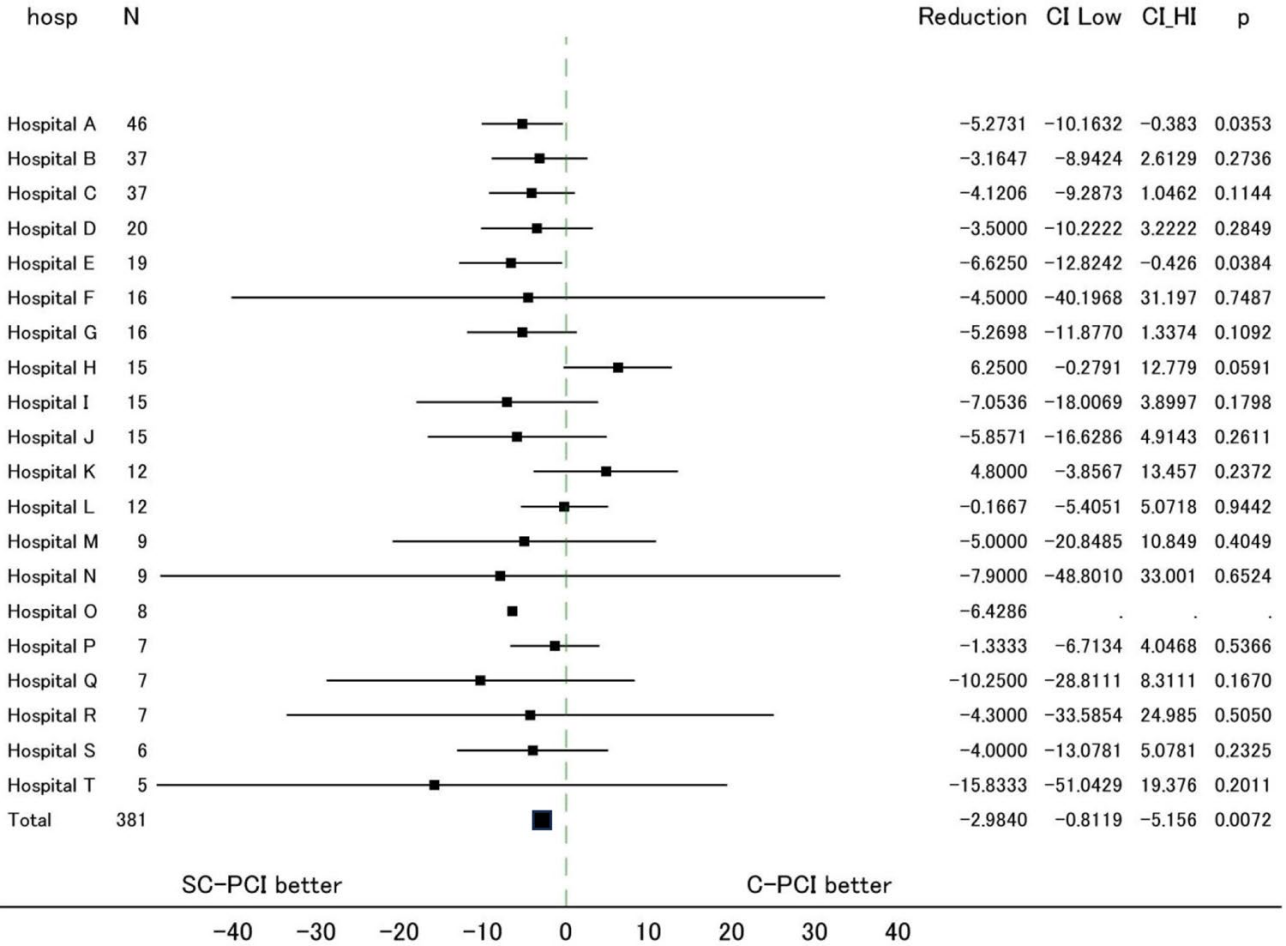
Analysis of sheath-to-balloon time by institute. Sheath-to-balloon time was separately analyzed for each institution in the SC-PCI method and the C-PCI method. *SC-PCI* single universal catheter percutaneous coronary intervention, *C-PCI* conventional percutaneous coronary intervention, *CI* confidence interval

The relationship between total number of catheters used in the procedure and S2B time is shown in Fig. 3. S2B time significantly decreases as the number of catheters used decreases (Number of catheters: 1 vs. 2 vs. 3 vs. 4 vs. 5 vs. 6; median S2B time: 13 min [interquartile range (IQR) 9–18], 13 min [IQR 9–23], 17 min [IQR 14–24], 24 min [IQR 20–34], 41 min [IQR 41–41], and 80 min [IQR 80–80], respectively, Fig. 3). Difference in S2B time by institution was analyzed from 20 institutes where at least 5 cases were enrolled in the study. As a result, S2B time of the SC-PCI method and the C-PCI method was consistently shorter in 18 out of 20 institutes (90%) (Fig. 4). On the other hand, consistent trend in D2B time was not found due to the wide variation among facilities.

### Clinical outcomes

The follow-up completion rate was 100% at 30 days and 99.5% at 1 year. The secondary endpoints at 30 days follow-up included all-cause death, cardiac death, and hemorrhage were similar between both groups. BARC 3 or 5 bleeding at PCI puncture site was zero in both groups. Other endpoints such as recurrent MI and stroke at 30 days were also comparable. Prevalence of all-cause mortality, recurrent MI, stroke, and bleeding at 1-year follow-up was also similar between the 2 groups (Table 5).

**Table 5:** Clinical outcome at 30 days and 1 year

	SC-PCI group *N* = 194	Conventional group *N *= 186	Total *N* = 380	p value
**30-day follow-up**				
All-cause death—no.(%)	5 (2.6)	5 (2.7)	10 (2.6)	0.94
Cardiac death—no.(%)	1 (0.5)	4 (2.2)	5 (1.3)	0.16
Non-cardiac death—no.(%)	4 (2.1)	1 (0.5)	5 (1.3)	0.19
Recurrent MI—no.(%)	0	0	0	
Stroke—no.(%)	3 (1.5)	2 (1.1)	5 (1.3)	0.69
Hemorrhagic—no.(%)	1 (0.5)	0	1 (0.3)	
Ischemic—no.(%)	2 (1.0)	2 (1.1)	4 (1.1)	
Bleeding (BARC 3 or 5)—no.(%)	3 (1.6)	1 (0.5)	4 (1.1)	0.69
Puncture site for PCI—no.(%)	0	0	0	
Puncture site for assist device- no.(%)	1 (0.5)	0	1 (0.3)	
Gastrointestinal tract—no.(%)	1 (0.5)	0	1 (0.3)	
Cardiac rupture—no.(%)	0	1 (0.5)	1 (0.3)	
Intracranial hemorrhage—no.(%)	1 (0.5)	0	1 (0.3)	
**1 year**				
All-cause mortality—no.(%)	8 (4.2)	7 (3.8)	15 (3.9)	0.86
Cardiac death—no.(%)	1 (0.5)	6 (3.3)	7 (1.8)	
Non-cardiac death—no.(%)	7 (3.7)	1 (0.5)	8 (2.1)	
Recurrent MI—no.(%)	2 (1.0)	0	2 (0.5)	0.55
Stroke—no.(%)	3 (1.5)	5 (2.8)	8 (2.1)	0.44
Hemorrhagic—no.(%)	1 (0.5)	3 (1.6)	4 (1.1)	
Ischemic—no.(%)	2 (1.0)	2 (1.1)	4 (1.1)	
Bleeding (BARC 3 or 5) – no.(%)	3(1.6)	3(1.7)	6(1.6)	0.96

*MI* myocardial infarction, *BARC* bleeding academic research consortium, *PCI* percutaneous coronary intervention

## Discussion

The SPEEDY-PCI trial is the first prospective, multicenter, randomized-controlled study to compare the efficacy of the SC-PCI method with the C-PCI method in STEMI patients undergoing primary PCI, predominantly via radial or distal-radial access (97% of cases). The main findings of the study were as follows: 1) S2B time, the primary endpoint of the study, was significantly shorter with the SC-PCI method compared to the C-PCI method. 2) Despite this reduction in procedural time, safety outcomes, including the incidence of coronary dissection, were comparable between the two groups, with no cases of coronary dissection observed in the SC-PCI method. 3) The SC-PCI method achieved a high success rate allowing for both diagnostic angiography and primary PCI with only a universal guiding catheter, while significantly reducing the total number of catheters used. 4) D2B time was similar between the SC-PCI method and the C-PCI method, with considerable variability between facilities.

The SC-PCI method resulted in a 3-min reduction in S2B time compared to the C-PCI method. This finding is consistent with the previous retrospective observational studies, which reported reductions ranging from 4 to 10 min [13, 14]. To reduce total ischemic time, several clinical studies have focused on shortening the time from symptom onset to hospital arrival and from the hospital door to the catheter lab [3–8]. However, there have been few studies specifically targeting the reduction of S2B time. In the current study, reducing the total number of catheters used with the SC-PCI method was one of the independent predictors for shorter S2B time along with hospitals and access change, suggesting the efficacy of the SC-PCI method. Although the observed 3-min reduction in S2B time might seem modest and D2B time remained unchanged, even small reductions in procedural time could potentially contribute to reduce infarct size and accelerating symptom relief in patients with STEMI.

Prior studies have reported greater time savings with other strategies, such as performing PCI directly before complete coronary angiography (CAG) [17–19], However, a recently published single-center RCT [17] highlighted a potential safety concern, showing a numerically higher prevalence of coronary dissection in the group undergoing PCI for the culprit lesion before completing CAG compared to the group where PCI followed complete CAG (6.7 vs. 2.1%, *p* = 0.13). Coronary artery dissection, particularly involving the left main coronary artery, is a significant complication of guiding catheters and can have serious consequences. In contrast, the current study demonstrated no worsening of any clinical endpoints. Furthermore, no coronary artery dissection was observed in patients using the IKARI curve, consistent with the previous reports [12, 13]. Dissections of the left main coronary artery associated with the IKARI curve remain extremely rare, further supporting the safety of this approach. While the Ikari curve is one of the most widely used guiding catheters not only in Japan but also, globally, further studies are needed to confirm whether other types of universal guiding catheters can provide comparable safety and procedural performance.

In this study, more than 97% of cases were performed using the radial or distal-radial approach, which was significantly higher than that reported in other registries or randomized-controlled studies of primary PCI [20, 21]. The success rate of the SC-PCI method with an Ikari curve was 88.1%, consistent with a previous study that reported a rate of 92.6%. In earlier retrospective observations of the SC-PCI method with 621 consecutive cases, the success rate using IL-3.5 guiding catheters was 98.1% [12], which is numerically higher than the SC-PCI method in primary PCI.

D2B time, another important aspect of reducing total ischemic time, was similar between the two groups in this study. This finding differs from previous studies. As the study was conducted during the COVID-19 era (November 2022 to October 2023), the door to catheter room time might have been extended, with considerable variability between facilities. Some facilities performed chest CT scans to determine if patients had COVID-19 pneumonia, while others waited for COVID-19 antigen test results. In-hospital mortality among STEMI patients was reported to be worse during the COVID-19 era [22, 23], and prolonged D2B time may have contributed to the increased mortality. Further investigation is needed to confirm these findings.

## Limitations

Some limitations of our study should be considered. First, although the sample size was initially calculated to be 400 patients, the inclusion of a higher expected number of patients with MI with MINOCA led to an increase in enrollment to 430 patients. Ultimately, 380 cases met the inclusion criteria for analysis.

Second, the current study was conducted in facilities where over 97% of cases utilized radial or distal-radial access for primary PCI. The IL guiding catheter was originally designed for radial access; thus, although routine radial access for primary PCI is recommended by guidelines [24, 25], the results may differ for physicians who are not accustomed to using the radial approach. However, given the increasing global adoption of radial access, the findings are likely relevant to many contemporary practice settings.

Third, it involved only operators experienced with the Ikari-Left guiding catheter. As operator experience is a key determinant of primary PCI success, these results may not be fully generalizable to less-experienced centers. Further studies could explore the learning curve associated with adopting the SC-PCI method.

Fourth, detailed lesion characteristics, such as the degree of calcification, bifurcation involvement, and procedural features, including the use of intravascular imaging or distal protection devices, were not systematically collected in this study, as the primary objective was to evaluate procedural time metrics (S2B and D2B) between the SC-PCI and C-PCI methods.

The last limitation is that the process of obtaining informed consent and confirming eligibility might have prolonged D2B time, potentially diluting the procedural time benefits observed.

## Conclusions

The SC-PCI method, utilizing the Ikari curve, demonstrated a meaningful reduction in PCI procedure time while maintaining safety and primary PCI success in this study. These findings suggest that SC-PCI method may be a feasible approach for streamlining the PCI process in STEMI patients and has the potential to serve as a first-line strategy among operators who are experienced in performing PCI with the IKARI curve. Further studies are warranted to confirm these results and assess their long-term clinical implications.

## Supplementary Information

Below is the link to the electronic supplementary material.

Supplementary file1 (DOCX 63 KB)

## Data Availability

The datasets generated and analyzed during the current study are available from the corresponding author upon reasonable request.
