# Supplementary material for: Single catheter primary percutaneous coronary intervention method in patients with ST-elevation myocardial infarction: the SPEEDY-PCI study
Source: Cardiovasc Interv Ther. 2025 Jul 7;40(4):807–19. doi: 10.1007/s12928-025-01162-1 (PMC12432066; doi:10.1007/s12928-025-01162-1)
Supplement: Supplementary file 1 — Supplementary file1 (DOCX 63 KB) [file 12928_2025_1162_MOESM1_ESM.docx]

**Supplemental Table**

**Supplemental Table 1. List of hospitals participated**

| Sapporo Cardio Vascular Clinic | Showa University Fujigaoka Hospital | Kindai University Hospital |
| --- | --- | --- |
| Hanaoka Seishu Memorial Hospital | Kanagawa Cardiovasculer and respiratory center | Osaka Rosai Hospital |
| Tochigi Medical Center | Shin-yurigaoka General Hospital | Kishiwada City Hospital |
| Kanazawa Cardiovascular Hospital | Higashiyamato Hospital | Tenri Hospital |
| Toyama Prefectural Central Hospital | Yamatoseiwa Hospital | Sakurakai Takahashi Hospital |
| Asama General Hospital | Sagamihara Kyodo Hospital | Veritas Hospital |
| Funabashi Municipal Medical Center | Ebina General Hospital | Hiroshima Prefectural Hospital |
| Ageo Central General Hospital | Tokai University Hospital | National Hospital Organization |
| Tokorozawa Heart Center | Hiratsuka Kyosai Hospital | Higashihiroshima Medical Center |
| The University of Tokyo Hospital | Hiratsuka City Hospital | Shuto General Hospital |
| Nihon University Itabashi Hospital | Fuji City General Hospital | Tokuyama Central Hospital |
| Sonoda Daiichi Hospital | Okamura Memorial Hospital | TakamatsuRed Cross Hospital |
| Showa University Hospital | Juntendo University Shizuoka Hospital | Harasanshin Hospital |
| Japanese Red Cross Musashino Hospital | Hamamatsu University Hospital | Kokura Memorial Hospital |
| Tokai University Hachioji Hospital | Toyota Memorial Hospital | Munakata Suikokai General Hospital |
| Mitsui Memorial Hospital | Kasugai Municipal Hospital | Saiseikai Kumamoto Hospital |
| Yokohama City University Medical Center | Ichinomiya Nishi Hospital | Tenyoukai Central Hospital |
| St.Marianna University Hospital | Gifu Heart Center |  |

**Supplemental Table 2. Procedural characteristics**

|  | SC-PCI group  N=194 | Conventional group  N=186 | Total  N=380 | p value |
| --- | --- | --- | --- | --- |
| Access site |  |  |  |  |
| Radial / Distal radial - no. (%) | 189 (97.4%) | 181 (97.3%) | 370 (97.4%) | 0.61 |
| Radial - no.(%) | 161 (83.0%) | 146 (78.5%) | 307 (80.8%) |  |
| Distal radial - no.(%) | 28 (14.4%) | 35 (18.8%) | 63 (16.6%) |  |
| Femoral - no.(%) | 4 (2.1%) | 3 (1.6%) | 7 (1.8%) |  |
| Other - no.(%) | 1 (0.5%) | 2 (1.1%) | 3 (0.8%) |  |
| Access change -no.(%) | 8 (4.1%) | 6 (3.2%) | 14 (3.7%) | 0.64 |
| Culprit lesion |  |  |  |  |
| RCA - no.(%) | 63 (32.5%) | 69 (37.1%) | 133 (35.0%) | 0.18 |
| LAD - no.(%) | 118 (60.8%) | 100 (53.8%) | 217 (57.1%) |  |
| LCX - no.(%) | 11 (5.7%) | 17 (9.1%) | 28 (7.4%) |  |
| LM - no.(%) | 2 (1.0%) | 0 | 2 (0.5%) |  |
| No. of vessel disease |  |  |  |  |
| 1 - no.(%) | 129 (66.5%) | 118 (63.4%) | 247 (65.0%) | 0.63 |
| 2 - no.(%) | 45 (23.2%) | 43 (23.1%) | 88 (23.2%) |  |
| 3 - no.(%) | 20 (10.3%) | 25 (13.4%) | 45 (11.8%) |  |
| Non-protected left main disease | 7 (3.6%) | 3 (1.6%) | 10 (2.6%) | 0.22 |
| No. of angiographic catheter | 0.09 ± 0.4 | 1.7 ± 0.6 | 0.9 ± 0.9 | <0.0001 |
| No. of guiding catheter | 1.1 ± 0.3 | 1.1 ± 0.4 | 1.1 ± 0.3 | 0.86 |
| No. of total catheter | 1.2 ± 0.6 | 2.7 ± 0.7 | 2.0 ± 1.0 | <0.0001 |
| Guiding catheter shape |  |  |  | <0.0001 |
| Ikari - no.(%) | 184 (94.9%) | 76 (40.9%) | 261 (68.7%) |  |
| Judkins - no.(%) | 6 (3.1%) | 44 (23.7%) | 49 (12.9%) |  |
| Amplatz - no.(%) | 1 (0.5%) | 10 (5.4%) | 11 (2.9%) |  |
| VODA/EBU/XB - no.(%) | 2 (1.0%) | 47 (25.2%) | 49 (12.9%) |  |
| Others - no.(%) | 1 (0.5%) | 9 (4.8%) | 10 (2.6%) |  |
| Sheath size |  |  |  | 0.48 |
| sheathless - no.(%) | 18 (9.3%) | 24 (12.9%) | 42 (11.0%) |  |
| 5F - no.(%) | 3 (1.6%) | 1 (0.5%) | 4 (1.1%) |  |
| 6F - no.(%) | 160 (82.5%) | 146 (78.5%) | 306 (80.5%) |  |
| 7F - no.(%) | 13 (6.7%) | 15 (8.1%) | 28 (7.4%) |  |
| Guiding catheter size |  |  |  |  |
| 5F - no.(%) | 2 (1.0%) | 1 (0.5%) | 3 (0.8%) | 0.32 |
| 6F - no.(%) | 184 (94.9%) | 171 (91.9%) | 355 (93.4%) |  |
| 7F - no.(%) | 8 (4.1%) | 14 (7.5%) | 22 (5.8%) |  |
| Guiding catheter shaft |  |  |  |  |
| Mach1 - no.(%) | 20 (10.3%) | 9 (4.8%) | 29 (7.6%) | 0.0009 |
| Heartrail2 - no.(%) | 115 (59.3%) | 92 (49.5%) | 207 (54.5%) |  |
| Launcher - no.(%) | 2 (1.0%) | 11 (5.9%) | 13 (3.4%) |  |
| Taiga - no.(%) | 3 (1.6%) | 4 (2.2%) | 7 (1.8%) |  |
| Profit - no.(%) | 9 (4.6%) | 2 (1.1%) | 11 (2.9%) |  |
| Hyperion - no.(%) | 45 (23.2%) | 67 (36%) | 112 (29.5%) |  |
| Others - no.(%) | 0 | 1 (0.5%) | 1 (0.3%) |  |
| Ikari curve size |  |  |  |  |
| IL30 - no./total no. (%) | 2/185 (1.1%) | 0/76 (0%) | 2/261 (0.8%) | 0.45 |
| IL35 - no./total no. (%) | 111/185 (60.0%) | 44/76 (58%) | 155/261 (59.3%) |  |
| IL375 - no./total no. (%) | 6/185 (3.2%) | 2/76 (2.6%) | 8/261 (3.1%) |  |
| IL40 - no./total no. (%) | 66/185 (35.7%) | 30/76 (39.5%) | 96/261 (36.8%) |  |
| IL45 - no./total no. (%) | 0 | 0 | 0 |  |
| Coronary stent implantation | 176 (90.8%) | 165 (88.7%) | 341 (89.7%) | 0.75 |
| Synergy stent - no.(%) | 159 (82.0%) | 144 (77.4%) | 303 (79.7%) |  |
| Other drug eluting stents - no.(%) | 17 (8.8%) | 21 (11.3%) | 38 (10.0%) |  |
| Intentional stentless - no.(%) | 15 (7.7%) | 18 (9.7%) | 33 (8.7%) |  |
| Stent delivery failure - no.(%) | 1 (0.5%) | 2 (1.1%) | 3 (0.8%) |  |
| Others - no.(%) | 2 (1.0%) | 1 (0.5%) | 3 (0.8%) |  |
| Post-PCI pacemaker - no.(%) | 3 (1.6%) | 4 (2.2%) | 7 (1.8%) | 0.66 |
| Post-PCI IABP - no.(%) | 10 (5.2%) | 8 (4.3%) | 18 (4.7%) | 0.70 |
| Post-PCI ECMO - no.(%) | 2 (1.0%) | 2 (1.1%) | 4 (1.1%) | 0.96 |
| Post-PCI Impella - no.(%) | 1 (0.5%) | 0 | 1 (0.3%) | 0.33 |
